# Correction: Risk factors for critical COVID-19 illness during Delta- and Omicron-predominant period in Korea; using K-COV-N cohort in the National health insurance service

**DOI:** 10.1371/journal.pone.0307925

**Published:** 2024-07-24

**Authors:** Kyung-Shin Lee, Min Jin Go, Youn Young Choi, Min-Kyung Kim, Jaehyun Seong, Ho Kyung Sung, Jaehyun Jeon, Hee-Chang Jang, Myoung-Hee Kim

The published funding statement is incorrect. The correct funding statement is:

This study was conducted as part of the public-private joint research on COVID-19 co-hosted by the KDCA and the NHIS. This study used the databases of the KDCA (Korea Disease Control and Prevention Agency, Republic of Korea) and the NHIS (National Health Insurance Service, Republic of Korea) for policy and academic research. The research number of this study is KDCA-NHIS-2022-1-527. This study was also supported by the Korea National Institute of Health (KNIH) research project (2022-NI-037-01). The funders had no role in study design, data collection and analysis, decision to publish, or preparation of the manuscript.
