# Supplementary material for: Risk factors for critical COVID-19 illness during Delta- and Omicron-predominant period in Korea; using K-COV-N cohort in the National health insurance service
Source: PLoS One. 2024 Mar 14;19(3):e0300306. doi: 10.1371/journal.pone.0300306 (PMC10939205; doi:10.1371/journal.pone.0300306)
Supplement: S1 Fig — (DOCX) [file pone.0300306.s001.docx]

Figure S1. Weekly epicurves stratified by age group for confirmed and critical COVID-19 cases

| 1. Confirmed cases   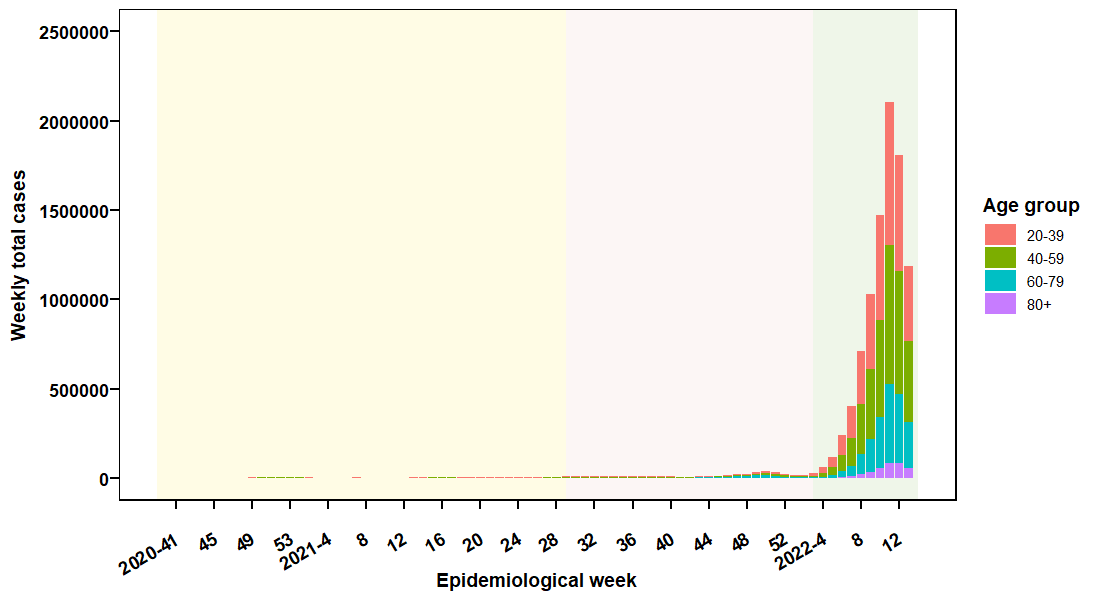 |
| --- |
| 1. Critical cases   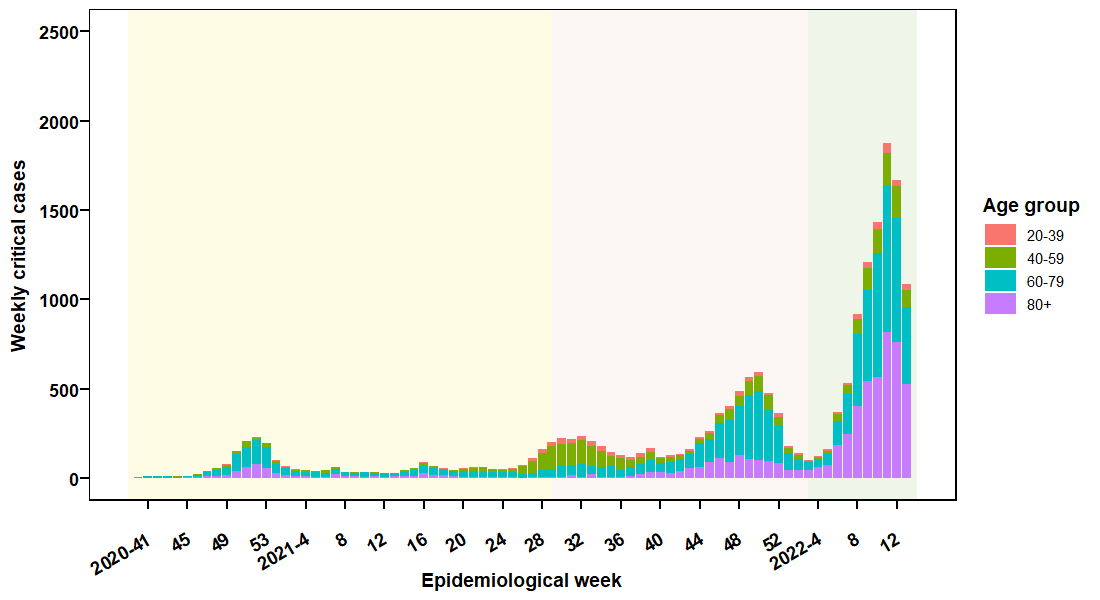 |

Note: We generated weekly epidemic curves stratified by age group for confirmed and critical cases of COVID-19 infection using the *ggplot2* package using R version 4.1.3 (R Development Core Team, https://cran.r-project.org/).
