# Supplementary material for: Risk factors for critical COVID-19 illness during Delta- and Omicron-predominant period in Korea; using K-COV-N cohort in the National health insurance service
Source: PLoS One. 2024 Mar 14;19(3):e0300306. doi: 10.1371/journal.pone.0300306 (PMC10939205; doi:10.1371/journal.pone.0300306)
Supplement: S2 Fig — (DOCX) [file pone.0300306.s002.docx]

Figure S2. Importance ranking of the characteristics of critical patients with COVID-19, according to the Gini index using random forest method

| (A) |
| --- |
| 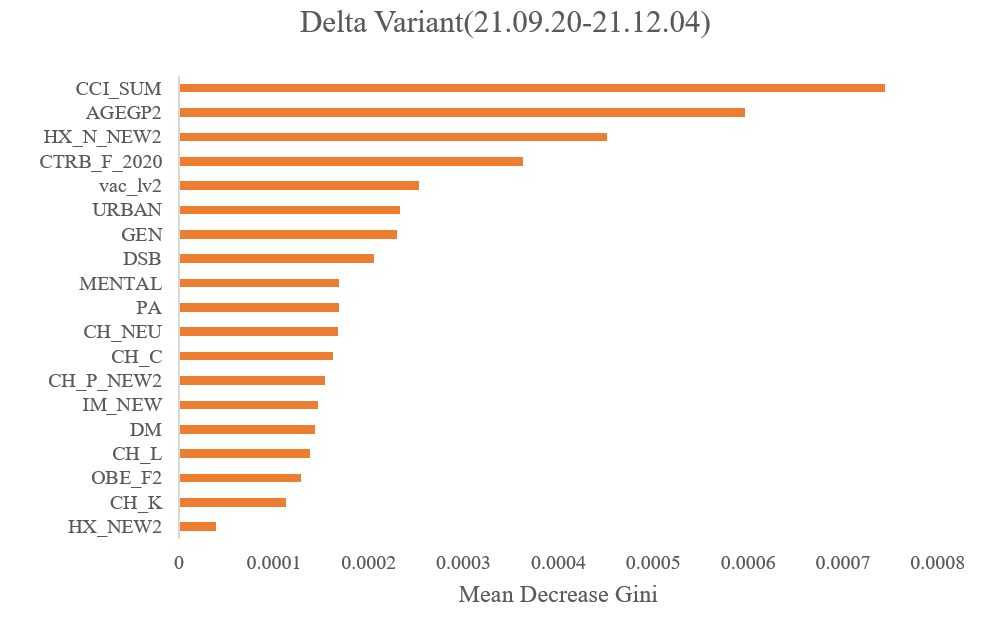 |
| Note: CCI_SUM; Charlson comorbidity index, AGEGP2; age group, HX_N_NEW2; number of underlying disease, CTRB_F_2020; Income level, vac_lv2; COVID-19 vaccination, URBAN; region, GEN; sex, DSB; Type of disability, MENTAL; mental disease, PA; physical activity, CH_NEU; Chronic neurologic disease, CH_C; Chronic cardiac disease, CH_P_NEW2; Chronic pulmonary disease, IM_NEW; Immunosuppression, DM; Diabetes mellitus, CH_L; Chronic liver disease, OBE_F2; Obesity, CH_K; Chronic kidney disease, HX_NEW2; Have diagnosed underlying disease. |
| (B) |
| 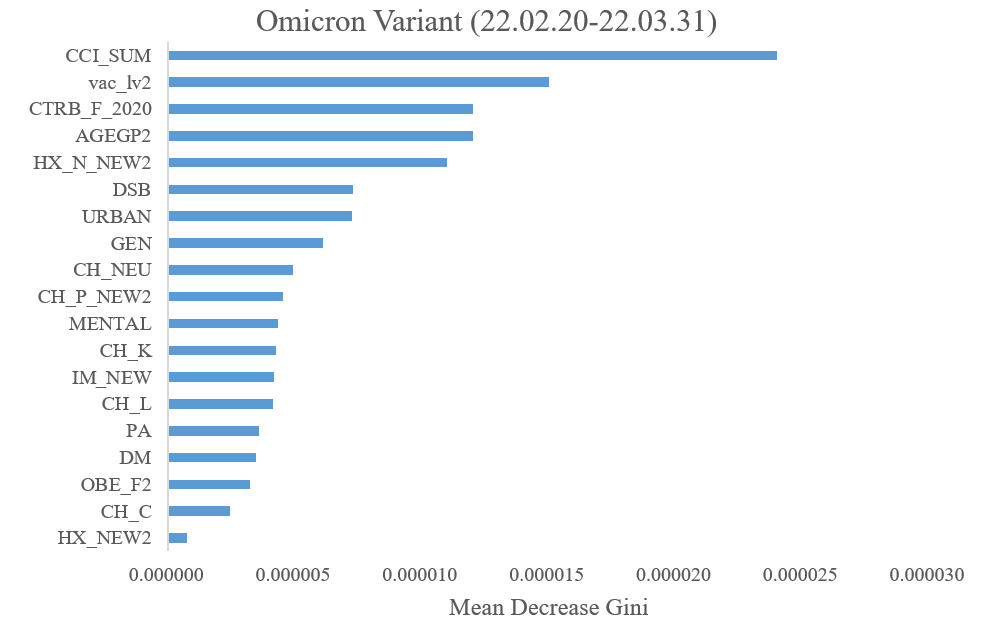 |

Note: CCI_SUM; Charlson comorbidity index, vac_lv2; COVID-19 vaccination, CTRB_F_2020; Income level, AGEGP2; age group, HX_N_NEW2; number of underlying diseases, DSB; Type of disability, URBAN; type of region, GEN; sex, CH_NEU; Chronic neurologic disease, CH_P_NEW2; Chronic pulmonary disease; MENTAL; Mental disease, CH_K; Chronic kidney disease, IM_NEW; Immunosuppression, CH_L; Chronic liver disease, PA; Physical activity, DM; Diabetes mellitus, OBE_F2: Obesity, CH_C; Chronic cardiac disease, HX_NEW2: have diagnosed underlying disease.
