# Supplementary material for: Risk factors for critical COVID-19 illness during Delta- and Omicron-predominant period in Korea; using K-COV-N cohort in the National health insurance service
Source: PLoS One. 2024 Mar 14;19(3):e0300306. doi: 10.1371/journal.pone.0300306 (PMC10939205; doi:10.1371/journal.pone.0300306)
Supplement: S1 Table — (DOCX) [file pone.0300306.s003.docx]

Table S1. List of population-based previous studies to investigate risk factors for COVID-19 severity.

1. Korean study

| No | First Author | Year | Journal name | Journal Vol. | Study Design | period | COVID Patients Included, No | Age  (Years) | Sex(% Female) | Outcome | Result(risk factors) | Stastical analysis | Adjusting factors |
| --- | --- | --- | --- | --- | --- | --- | --- | --- | --- | --- | --- | --- | --- |
| 1 | Yoo-Yeon Kim | 2023 | J Korean Med Sci | 20;38(11):e87 | Retrospective study | **July 2021–January 2022** | 530,827 | 12 years or older | 269,805(50.8%) | Severe disease and death | Three doses of **mRNA vaccines (aOR, 0.05; 95% confidence interval [CI], 0.04-0.07), two doses of viral vector vaccine followed by an additional dose of mRNA vaccine (aOR, 0.06; 95% CI, 0.05–0.07), and one dose of viral vector vaccine followed by two doses of mRNA vaccines (aOR, 0.08; 95% CI, 0.03–0.15)** were associated with lower risks of severe disease | multivariable logistic regression model | composite of sex, age categories, area of residence, month of diagnosis, and vaccination status |
| 2 | Suyoung Jo | 2022 | PLOS ONE | 17(8) | observational, retrospective cohort study | **~30, April 2020** | 5,153 | 0~80+ | 3,017 (58.5) | The effect on in-hospital mortality | Factor 1 symptoms were cold-like symptoms, factor 2 were **neurological and gastrointestinal symptoms/ Factor 1 (HR 1.14, 95% confidence interval [95% CI] 1.01–1.30) and factor 3 (HR 1.25, 95% CI 1.19–1.31) were associated with a higher risk for death** | Cox proportional hazards model | age group, sex, obesity, heart rate, and comorbidities |
| 3 | Hye Jin Shi | 2022 | J Korean Med Sci | 37(22) | retrospective study, multicenter | **July 1, 2021-November 30, 2021** | 448 | 60.18 ± 16.51 | 100 (44.4) | Clinical Characteristics and Risk Factors for Mortality | Age (odds ratio [OR], 7.902; 95% confidence interval [CI], 2.754–18.181), mechanical ventilation therapy (OR, 17.233; 95% CI, 8.439–35.192), highest creatinine > 1.5 mg/dL (OR, 17.631; 95% CI, 8.321–37.357), and combined blood stream infection (OR, 7.092; 95% CI, 1.061–18.181) were identified as independent predictors of mortality in total patients. | Logistic regression analysis | age, Underlying diseases, Initial manifestation, Initial laboratory findings, MV, ECMO, CRRT, Complication |
| 4 | Yong Jun Choi | 2021 | PLOS ONE | 16(7) | retrospective study | **~May 15, 2020** | 7,590 | 0~70+ | 4495(59.2%) | mortality, ICU admission, affecting duration of hospital stay, influencing total hospital cost | Mortality: congestive heart failure (OR, 1.724; 95% CI, 1.211–2.456; P = 0.003), dementia (OR, 1.598; 95% CI, 1.108–2.305;P = 0.012), **diabetes with or without chronic complications** (OR, 1.821; 95% CI, 1.255–2.644; P = 0.002 and OR, 1.518; 95% CI, 1.063–2.169; P = 0.022, respectively**), renal disease** (OR,2.299; 95% CI, 1.371–3.858; P = 0.002), and/or malignancy (OR,1.529; 95% CI, 1.022–2.257;P = 0.039) **ICU admission: diabetes with chronic complications** (OR, 1.811; 95% CI, 1.241–2.642; P = 0.002) | Logistic regression analysis | age, sex, concomitant chronic conditions |
| 5 | Wonjun Ji | 2020 | J Korean Med Sci | 29;35(25):e237 | nationwide, retrospective case-control study | **~May 15, 2020** | 7,341 | aged ≥ 18 years | 4371(59.5%) | Infection and Severity of COVID-19 | Severity of COVID-19 : diabetes (OR, 1.247; 95% confidential interval, 1.009–1.543), **hypertension (ORR, 1.245–1.317), chronic lower respiratory disease (ORR, 1.216–1.233), chronic renal failure, and end-stage renal disease** | Logistic regression analysis | age, sex, region, healthcare utilization,insurance status |

1. Another country study

| No | First Author | Year | Journal name | Journal Vol. | Study Design | Country | period | COVID Patients Included, No | Age(Years) | Sex(% Female) | Outcome | Result(risk factors) | Stastical analysis | Adjusting factors |
| --- | --- | --- | --- | --- | --- | --- | --- | --- | --- | --- | --- | --- | --- | --- |
| 1 | Christina YEK et al. | 2022 | Morbidity and Mortality Weekly Report | 71 | retrospective | US | **2020.3.~2021.10.** | 2,246 | 18+ | 1,294  (57.6%) | severe disease | age ≥65 years, immunosuppression, diabetes, and chronic kidney, cardiac, pulmonary, neurologic, and liver disease | Logistic regression analysis | age, sex, days since primary vaccination series completion, variant predominance, vaccine type, race |
| 2 | U Agrawal et al. | 2022 | The lancet | 400 | prospective cohorts | UK, Northern Ireland, Scotland, Wales | **2020.12.08.~2022.02.28.** | 16,208,600 | 18+ | 8,468,280(52.2%) | Severe COVID-19 (COVID-19-related hospitalisation or death) | Older adults (≥80 years vs 18–49 years; aRR 3·60 [95% CI 3·45–3·75]), those with comorbidities (≥5 comorbidities vs none; 9·51 [9·07–9·97]), being male (male vs female; 1·23 [1·20–1·26]), and those with certain underlying health conditionsin particular, individuals receiving immunosuppressants (yes vs no; 5·80 [5·53–6·09])—and those with chronic kidney disease (stage 5 vs no; 3·71 [2·90–4·74]) remained at high risk despite the initial booster. | Logistic regression analysis | race, socioeconomic deprivation status, |
| 3 | C Menni et al. | 2022 | The lancet | 399 | prospective cohorts | UK | **2021.6.1.~2022.01.17** | 63,002 | 18+ | 18,709(64%) | Hospitalisation/symptoms | 1. Sore throat, hoarse voice: omicron infection,  2. else symptoms delta infection 3. hospitalization: vaccination status (2 vs 3 doses) 4.duration of acute symptoms :delta 5. odds of recovering: delta | Logistic regression analysis | age, sex, presence of comorbidities , vaccination status |
| 4 | T Nyberg et al. | 2022 | The lancet | 399 | retrospective | UK | **2021.11.29.~2022.1.9.** | 4,135,347 | 0~80+ | 807,595(53.2%) | hospitalisation, death | Omicron versus delta HR estimates were lower for hospital admission (0·30 [0·28–0·32]) in unvaccinated cases than the corresponding HR estimated for all cases in the primary analysis. Booster vaccination with an mRNA vaccine was highly protective against hospitalisation and death in omicron cases (HR for hospital admission 8–11 weeks post-booster vs unvaccinated:0·22 [0·20–0·24] | Cox proportional hazards regression | sex, index of multiple deprivation decile, evidence of a previous infection, and year of age within each age band |
| 5 | TKT Lo et al. | 2022 | medrxiv | - | A population-based case-control study | Canada-Alberta | **2022.1.1~1.31.** | 90,989 | 18+ | 51,435(56.53%) | severe acute COVID-19 outcomes(hospitalization, intensive-care unit (ICU) admission, or death) | The highest aOR in the 70–79 age group (28.32; 95% CI 20.6–38.9) or among ≥80 years old (29.8; 21.6–41.0), males (1.4; 1.3–1.6); unvaccinated (16.1; 13.8–18.8), or patients with ≥3 underlying conditions (13.1; 10.9–15.8). | Logistic regression analysis | age, sex, number of underlying conditions, and vaccination status |
| 6 | J Skarbinski et al. | 2022 | The Lancet Regional Health - Americas | 12 | retrospective | US, UK, South africa | **2021.12.18-2022.1.7.** | 118,078 | 0~80+ | 64,416(55%) | hospitalization, respiratory support, death | The risk of hospitalization was higher among unvaccinated persons (aHR 8.34; CI 7.25-9.60) and those who completed a primary COVID-19 vaccination series (aHR 1.72; CI 1.49-1.97) compared with those who completed a primary vaccination series and an additional dose. The strongest risk factors for all severe clinical outcomes were older age, higher body mass index and select comorbidities. | multivariable Poisson regression | age, concomitant infections |
| 7 | P Bager et al. | 2021 | The lancet infectious diseases | 21 | observational cohort(prospective) | denmark | **2021.1.1.~2021.3.24.** | total: 50958 hospitalization: 1942 | 0~60+ | 942(48.5%) | hospitalization | For a similar comparison by vaccination status, the RR of hospitalisation was 0·57 (0·44–0·75) among cases with no or only one dose of vaccine, 0·71 (0·60–0·86) among those who received two doses, and 0·50 (0·32–0·76) among those who received three doses. | multivariable Poisson regression | sex, age, calendar time, region, and comorbidities |
