# Supplementary material for: Risk factors for critical COVID-19 illness during Delta- and Omicron-predominant period in Korea; using K-COV-N cohort in the National health insurance service
Source: PLoS One. 2024 Mar 14;19(3):e0300306. doi: 10.1371/journal.pone.0300306 (PMC10939205; doi:10.1371/journal.pone.0300306)
Supplement: S2 Table — (DOCX) [file pone.0300306.s004.docx]

Table S2. ICD-10 Codes used to identify underlying conditions

| Underlying Condition | ICD-10 Codes |
| --- | --- |
| Any | Answer to have underlying diseases including obesity, diabetes mellitus, immunosuppression, chronic kidney disease, chronic neurological disease, chronic cardiac disease, chronic pulmonary disease, chronic liver disease, and mental disease |
| Underweight | answered BMI is under 18.5 in health examination between 2018 and 2020 |
| Overweight | answered BMI is between 23 and 25 in health examination between 2018 and 2020 |
| Obesity | E66x, Z68·25-Z68·29, Z68·3, Z68·4  or answered BMI is 25·0 and more in health examination between 2018 and 2020 |
| Diabetes mellitus | E10x, E11x, E13x, Z79·4, Z79·84 |
| Immunosuppression | Cxx, T86x, Z94·0-Z94·4, Z94·8, Z94·9, M05, M06, M07, M08, M32, M33, M45, G35, K50, K51, L95, M300, M340, M341, M348, M349, M350, M351, M352, M353, L405, I776, D510, D591, D6938, D70x, D71x, D80x, D81x, D82x, D83x, D84x, D89x |
| Cancer | Cxx |
| Solid organ or hematopoietic  stem cell transplantation | T86x, Z94·0-Z94·4, Z94·8, Z94·9 |
| Autoimmunity disease | M05, M06, M07, M08, M32, M33, M45, G35, K50, K51, L95, M300, M340, M341, M348, M349, M350, M351, M352, M353, L405, I776, D510, D591, D6938 |
| Immunodeficiency | D70x, D71x, D80x, D81x, D82x, D83x, D84x, D89x |
| Chronic kidney disease | I12x, I13x, K76·7, N18x, P96·0, Z99·2 |
| Chronic neurological disease | F01x, F02x, F03x, G31·0, G31·83, I69x, Z86·73, G70x, G71x, G72x, G73x, G04·1, G11·4, G80x, G81x, G82x, G83x |
| Dementia | F01x, F02x, F03x, G31·0, G31·83 |
| Cerebrovascular disease | I69x, Z86·73 |
| Myopathies | G70x, G71x, G72x, G73x |
| Paralytic syndromes | G04·1, G11·4, G80x, G81x, G82x, G83x |
| Chronic cardiac disease | I25x, Z95·1, I11·0, I27x, I42x, I43x, I50x, P29·0, I05x, I06x, I07x, I08x, I09x, I34x, I35x, I36x, I37x, I38x, I39x, Q23·0-Q23·3, Z95·2-Z95·4,I44x, I45x, I47x, I48x, I49x, Z95·0, I10-I15 |
| Coronary artery disease | I25x, Z95·1 |
| Heart failure and  cardiomyopathies | I11·0, I27x, I42x, I43x, I50x, P29·0 |
| Valvular heart disease | I05x, I06x, I07x, I08x, I09x, I34x, I35x, I36x, I37x, I38x, I39x, Q23·0-Q23·3, Z95·2-Z95·4 |
| Arrhythmias | I44x, I45x, I47x, I48x, I49x, Z95·0 |
| Hypertension | I10-I15 |
| Chronic pulmonary disease | J45x, J60x, J61x, J62x, J63x, J64x, J65x, J66x, J67x, J68·4, J70·1, J70·3, J84x, J47x, F17x, O99·33, T65·2, Z72·0, Z87·891, A15x, Z99·1, Z96·1 |
| Chronic obstructive pulmonary  disease | J43x, J44x |
| Asthma | J45x |
| Interstitial lung disease | J60x, J61x, J62x, J63x, J64x, J65x, J66x, J67x, J68·4, J70·1, J70·3, J84x |
| Bronchiectasis | J47x |
| Smoking history (current) | F17x, O99·33, T65·2, Z72·0, Z87·891 |
| Pulmonary tuberculosis | A15x |
| Long-term oxygen therapy | Z99·1,Z96·1 |
| Chronic liver disease | K71·7, K72·1, K74·3, K74·4, K74·6, K76·6, K76·0,K75·8, K70x, K75·4 |
| Cirrhosis | K71·7, K72·1, K74·3, K74·4, K74·6, K76·6 |
| Non-alcoholic fatty liver disease | K76·0, K75·8 |
| Alcoholic liver disease | K70x |
| Autoimmune hepatitis | K75·4 |
| Mental disease | F2x, F3x |
| Psychotic disorder | F2x |
| Mood disorder | F3x |
| Lack of physical activity | The group of never exercised, including muscle strengthening activity or moderate or vigorous intensity activity in health examination between 2018 and 2020. |
