# Supplementary material for: Risk factors for critical COVID-19 illness during Delta- and Omicron-predominant period in Korea; using K-COV-N cohort in the National health insurance service
Source: PLoS One. 2024 Mar 14;19(3):e0300306. doi: 10.1371/journal.pone.0300306 (PMC10939205; doi:10.1371/journal.pone.0300306)
Supplement: S3 Table — (DOCX) [file pone.0300306.s005.docx]

Table S3. Result of logistic regression analysis for severe or critical COVID-19 illness

|  | Delta-predominant  (921/20–12/21/04) | | Omicron-Predominant  (2/22/20–3/22/31) | | Scale of ORs  with color |
| --- | --- | --- | --- | --- | --- |
|  | Univariate model | Multivariate model^1^ | Univariate model | Multivariate model^1^ | NS |
|  | OR (95% CI) | aOR (95% CI) | OR (95% CI) | aOR (95% CI) | <1·0 |
| Sex |  |  |  |  | 1·0–5·0 |
| Male | 1 | 1 | 1 | 1 | 5·1–9·9 |
| Female | 0·61 (0·56, 0·66) | 0.79 (0.75, 0.82) | 0·35 (0·33, 0·37) | 0.95 (0.93, 0.97) | 10·0≤ |
| Age categories, year |  |  |  |  |  |
| 20–39 | 1 | 1 | 1 | 1 |  |
| 40–59 | 1·93 (1·79, 2·08) | **2.18 (2.02, 2.36)** | 1·62 (1·58, 1·67) | 1.14 (1.10, 1.19) |  |
| 60–79 | 4·78 (4·46, 5·11) | **5.72 (5.27, 6.20)** | 6·01 (5·86, 6·16) | **2.17 (2.09, 2.26)** |  |
| 80+ | 14·54 (13·46, 15·71) | **12.63 (11.42, 13.96)** | 34·18 (33·34, 35·03) | **5.26 (5.02, 5.52)** |  |
| Vaccination |  |  |  |  |  |
| None | 1 | 1 | 1 | 1 |  |
| 1^st^ Dose | 0.30 (0.28, 0.31) | **0.42 (0.40, 0.45)** | 0.49 (0.47, 0.51) | 0.56 (0.52, 0.60) |  |
| More than 2^nd^ Dose | 0.32 (0.30, 0.33) | **0.16 (0.15, 0.17)** | 0.17 (0.16, 0.17) | 0.20 (0.20, 0.21) |  |
| Region |  |  |  |  |  |
| Rural | 1 | 1 | 1 | 1 |  |
| Urban | 1·06 (1·01, 1·10) | 0.94 (0.90, 0.99) | 1·12 (1·10, 1·13) | 0.96 (0.94, 0.98) |  |
| Unknown | 0·25 (0·19, 0·33) | 0.33 (0.25, 0.44) | 0·58 (0·50, 0·67) | 0.72 (0.43, 1.21) |  |
| Type of disability |  |  |  |  |  |
| No disability | 1 | 1 | 1 | 1 |  |
| Mild disability | 3·09 (2·87, 3·32) | 1.20 (1.11, 1.31) | 5·39 (5·27, 5·51) | 1.25 (1.20, 1.29) |  |
| Severe disability | 4·00 (3·68, 4·35) | 1.77 (1.60, 1.96) | 8·33 (8·15, 8·52) | 1.85 (1.77, 1.94) |  |
| Income level in 2020 |  |  |  |  |  |
| Medicaid | 1 | 1 | 1 | 1 |  |
| T1(Poor) | 0·38 (0·35, 0·41) | - | 0·22 (0·21, 0·23) | 0.92 (0.87, 0.97) |  |
| T2 | 0·33 (0·31, 0·36) | - | 0·19 (0·18, 0·19) | 0.93 (0.88, 0.97) |  |
| T3 (Rich) | 0·41 (0·38, 0·45) | - | 0·22 (0·22, 0·23) | 0.88 (0.84, 0.92) |  |
| Unknown | 0·17 (0·14, 0·20) | - | 0·16 (0·16, 0·17) | 0.93 (0.85, 1.02) |  |
| Underlying Condition |  |  |  |  |  |
| Yes*** | 2·33 (2·24, 2·44) | NA^2^ | 3·24 (3·18, 3·29) | NA^2^ |  |
| Underweight | NA | NA | 5.96 (4.55, 7.79) | 1.14 (1.07, 1.20) |  |
| Overweight | NA | NA | 4.88 (3.26, 7.31) | 0.85 (0.78, 0.93) |  |
| Obesity | 1·22 (1·16, 1·27) | 1.48 (1.41, 1.57) | 3.14 (2.18, 4.52) | 1.01 (0.94, 1.09) |  |
| Diabetes mellitus | 2·88 (2·76, 3·00) | 1.30 (1.24, 1.37) | 4·10 (4·04, 4·16) | 1.23 (1.20, 1.26) |  |
| Immunosuppression | 1·91 (1·82, 2·01) | NA^2^ | 3·08 (3·03, 3·13) | NA^2^ |  |
| Cancer | 2·14 (2·00, 2·28) | 1.13 (1.05, 1.21) | 4·26 (4·18, 4·33) | 2.28 (2.22, 2.34) |  |
| Solid organ or  hematopoietic stem  cell transplantation | 4·92 (3·79, 6·39) | **2.58 (1.91, 3.49)** | 7·92 (7·37, 8·52) | 2.55 (2.28, 2.85) |  |
| Autoimmunity disease | 1·55 (1·45, 1·66) | - | 1·77 (1·73, 1·81) | - |  |
| Immunodeficiency | 2·63 (2·11, 3·27) | - | 5·95 (5·64, 6·27) | 2.00 (1.86, 2.16) |  |
| Chronic kidney disease | 3·97 (3·68, 4·29) | 1.35 (1.23, 1.47) | 8·29 (8·11, 8·47) | 1.61 (1.56, 1.68) |  |
| Chronic neurological disease | 4·94 (4·67, 5·23) | NA^2^ | 12·9 (12·70, 13·11) | NA^2^ |  |
| Dementia | 5·35( 4·98, 5·75) | 1.31 (1.20, 1.43) | 13·79 (13·53, 14·06) | 1.52 (1.46, 1.58) |  |
| Cerebrovascular disease | 4·60 (4·22, 5·02) | 1.14 (1.02, 1.26) | 10·36 (10·13, 10·59) | 1.21 (1.16, 1.26) |  |
| Myopathies | 2·15 (1·62, 2·85) | - | 2·76 (2·54, 3·01) | - |  |
| Paralytic syndromes | 5·39 (4·91, 5·92) | 1.50 (1.34, 1.68) | 14·60 (14·28, 14·92) | 2.37 (2.27, 2.48) |  |
| Chronic cardiac disease | 3·51 (3·37, 3·67) | NA^2^ | 6·42 (6·32, 6·52) | NA^2^ |  |
| Coronary artery disease | 3·25 (3·00, 3·51) | 1.20 (1.10, 1.32) | 5·25 (5·13, 5·38) | 1.16 (1.11, 1.20) |  |
| Heart failure and cardiomyopathies | 3·31 (3·12, 3·51) | - | 8·11 (7·98, 8·24) | 1.41 (1.37, 1.46) |  |
| Valvular heart disease | 2·87 (2·43, 3·40) | - | 5·84 (5·59, 6·09) | 1.22 (1.14, 1.31) |  |
| Arrhythmias | 2·48 (2·31, 2·65) | - | 4·70 (4·61, 4·79) | 1.21 (1.17, 1.25) |  |
| Hypertension | 3·43 (3·29, 3·58) | 1.31 (1.24, 1.39) | 6·20 (6·11, 6·30) | 1.34 (1.31, 1.38) |  |
| Chronic pulmonary disease | 1·80 (1·72, 1·88) | NA^2^ | 1·83 (1·81, 1·86) | NA^2^ |  |
| Chronic obstructive pulmonary disease | 1·73 (1·66, 1·81) | 1.14 (1.09, 1.20) | 2·36 (2·33, 2·40) | 1.26 (1.23, 1.29) |  |
| Asthma | 1·54 (1·47, 1·61) | 1.11 (1.05, 1.17) | 1·84 (1·81, 1·87) | 1.15 (1.12, 1.17) |  |
| Interstitial lung disease | 4·24 (3·53, 5·09) | 1.68 (1.37, 2.06) | 7·83 (7·45, 8·23) | 1.97 (1.83, 2.11) |  |
| Bronchiectasis | 1·98 (1·71, 2·29) | - | 4·26 (4·10, 4·43) | 1.23 (1.16, 1.30) |  |
| Smoking history  (current) | 0·95 (0·44, 2·05) | - | 0·60 (0·58, 0·62) | 1.16 (1.12, 1.20) |  |
| Pulmonary tuberculosis | 2·58 (1·94, 3·44) | - | 6·74 (6·29, 7·23) | 1.56 (1.39, 1.75) |  |
| Long-term oxygen  therapy | 2·29 (2·12, 2·48) | 1.11 (1.01, 1.21) | 3·05 (2·98, 3·13) | 1.05 (1.01, 1.08) |  |
| Chronic liver disease | 1·51 (1·44, 1·60) | NA^2^ | 1·47 (1·44, 1·49) | NA^2^ |  |
| Cirrhosis | 2·95 (2·53, 3·44) | 1.41 (1.19, 1.68) | 4·62 (4·40, 4·84) | 1.45 (1.35, 1.56) |  |
| Non-alcoholic fatty  liver disease | 1·42 (1·34, 1·51) | - | 1·26 (1·24, 1·29) | 0.90 (0.88, 0.93) |  |
| Alcoholic liver disease | 1·55 (1·40, 1·72) | - | 1·92 (1·85, 1·99) | 1.08 (1.02, 1.13) |  |
| Autoimmune hepatitis | 1·69 (1·03, 2·76) | - | 2·32 (2·01, 2·68) | - |  |
| Mental disease | 2·60 (2·48, 2·72) | NA^2^ | 4·81 (4·74, 4·88) | NA^2^ |  |
| Psychotic disorder | 3·09 (2·81, 3·39) | 1.26 (1.12, 1.41) | 5·81 (5·65, 5·96) | 1.54 (1.47, 1.62) |  |
| Mood disorder | 2·56 (2·45, 2·68) | 1.20 (1.13, 1.27) | 4·70 (4·63, 4·77) | 1.42 (1.38, 1.45) |  |
| Lack of physical activity | 1.47 (1.41, 1.53) | 1.27 (1.21, 1.34) | 2.20 (2.17, 2.24) | 1.25 (1.22, 1.28) |  |
| Charlson comorbidity index in 2020 (Continuous) | 1·30 (1·29, 1·31) | NA^2^ | 1·44 (1·44, 1·45) | NA^2^ |  |
| Number of underlying disease(Continuous)^3^ | 1·40(1·39, 1·42) | NA^2^ | 1·65 (1·65, 1·66) | NA^2^ |  |

Note: OR, odds ratio; aOR, adjusted odds ratio; CI, confidence interval; NS, Non-significant; NA, Not analysis

^1^After selecting variables using stepwise regression

^2^These variables weren’t included in stepwise regression model to overcome multicollinearity.

^3^ Have diagnosed and number of underlying diseases including obesity, diabetes mellitus, immunosuppression, chronic kidney disease, chronic neurological disease, chronic cardiac disease, chronic pulmonary disease, chronic liver disease, and mental disease
