# Supplementary material for: Risk factors for critical COVID-19 illness during Delta- and Omicron-predominant period in Korea; using K-COV-N cohort in the National health insurance service
Source: PLoS One. 2024 Mar 14;19(3):e0300306. doi: 10.1371/journal.pone.0300306 (PMC10939205; doi:10.1371/journal.pone.0300306)
Supplement: Attachment — Submitted filename: Review Comments to the Author_231211_f.docx [file pone.0300306.s006.docx]

Response to the Editor and Reviewers for Manuscript: PONE-D-23-23867

Thank you for providing us an opportunity to improve the quality of our paper. We have revised the manuscript according to the reviewers’ valuable and helpful comments, and have provided our responses to each comment as follows.

Review Comments to the Author
Please use the space provided to explain your answers to the questions above. You may also include additional comments for the author, including concerns about dual publication, research ethics, or publication ethics. (Please upload your review as an attachment if it exceeds 20,000 characters)

Reviewer #1: Since the therapy and vaccine coverage were significantly different throughout the Delta and Omicron periods, it is very challenging to compare the results of these two time periods.
Please think about including the subject count in the abstract since some readers may only want a quick summary of the study's findings without opening the complete text.

- Thanks for your comment. As you suggested, we included the statement in the abstract as follows.

**Before revision**

**Results:** During the Delta as well as the Omicron period, old age (≥80 years) showed the largest effect size among risk factors for critical COVID-19 illness (aOR = 18.08; 95% confidence interval [CI] = 14.71–22.23 for the Delta; aOR = 24.07; 95% CI = 19.03–30.44 for the Omicron period). We found that patients with solid organ transplant (SOT) recipients, unvaccinated, and interstitial lung disease had more than a two-fold increased risk of critical COVID-19 outcomes between the Delta and Omicron periods. However, risk factors such as urban residence, underweight, and underlying medical conditions, including chronic cardiac diseases, immunodeficiency, and mental disorders, had different effects on the development of critical COVID-19 illness between the Delta and Omicron periods.

**After revision**

**Results:** We analyzed 137,532 and 8,294,249 cases of COVID-19 infection during the Delta and the Omicron variant dominant period, respectively. During the Delta as well as the Omicron period, old age (≥80 years) showed the largest effect size among risk factors for critical COVID-19 illness (aOR = 18.08; 95% confidence interval [CI] = 14.71–22.23 for the Delta; aOR = 24.07; 95% CI = 19.03–30.44 for the Omicron period). We found that patients with solid organ transplant (SOT) recipients, unvaccinated, and interstitial lung disease had more than a two-fold increased risk of critical COVID-19 outcomes between the Delta and Omicron periods. However, risk factors such as urban residence, underweight, and underlying medical conditions, including chronic cardiac diseases, immunodeficiency, and mental disorders, had different effects on the development of critical COVID-19 illness between the Delta and Omicron periods.

Line 135: An individual was considered to have died from COVID-19 within a month of being diagnosed. Do you have a patient whose stay will be more than one month? What use does having this operational definition serve?

- As you pointed out, it is not clear whether a death that occurs after a COVID-19 infection is due to the infection itself. However, Joen et al (2023) reported that COVID-19 death numbers calculated by the operational definition of 30 days within infection did not significantly differ from the numbers extracted from the National Death Statistics with the information on cause of death. We added the following sentence in the methods as follows.

**Before revision**

**Method:** The fatality was defined as death within a month after COVID-19 diagnosis.

**After revision**

**Methods:** The fatality was defined as death within a month after COVID-19 diagnosis. We selected the 30-day period from infection to death, which was used as a reasonable proxy variable for COVID-19 fatalities verified in previous study (1).

Jeon, Jeangeun, et al. "Unequal burdens of COVID-19 infection: a nationwide cohort study of COVID-19-related health inequalities in Korea." *Epidemiology and Health* 45 (2023).

Lines 270–274: The author can explain these data in the discussion section, but it would be better to include them in the results section.

- As you suggested, we deleted these sentence in the discussion. Indeed, those findings were already included in the Results section.

**Before revision**

**Discussion (Line 270-274)**

The proportion of patients with critical illness was approximately 1.9% and 0.1%, and the proportion of patients with grade 7 severity (treated with ECMO or mechanical ventilation and continuous venous hemodialysis) was 4.9% and 1.5%, during the Delta and Omicron period, respectively. Patients aged 80 or over accounted for 6% and 4.1% of all cases in the Delta and Omicron period, respectively. However, these numbers accounted for 26.5% and 44.1% of all critical cases respectively, indicating that a high proportion of the elderly population was susceptible to critical COVID-19 illness.

**After revision**

Deleted

Lines 306-311: The explanation does not fit the assertion..... For those patients, COVID-19 symptoms are exacerbated by uncontrolled and dysregulated immunity (39, 40), and respiratory tract defects may impair the immune response to infection, resulting in susceptibility to SARS-CoV-2, virus spread, pulmonary disease, and systemic inflammation.

- As your commented, we modified the paragraph and added reference as follows.

**Before revision**

We found that solid organ transplant (SOT) recipients had more than a two-fold increase in risk for severe COVID-19 outcomes, which supports the observations made in previous studies (37, 38). For those patients, COVID-19 symptoms are exacerbated by uncontrolled and dysregulated immunity (39, 40), and respiratory tract defects may impair the immune response to infection, resulting in susceptibility to SARS-CoV-2, virus spread, pulmonary disease, and systemic inflammation.

**After revision**

Solid organ transplant (SOT) recipients had more than a two-fold increase in risk for severe COVID-19 outcomes, which supports the observations made in previous studies (37-39). It revealed that the higher the type and dose of immunosuppressants, the more susceptible the transplanted organ, such as the heart or lungs, the higher the risk of serious illness if the patient is older or has an underlying disease (38-39). Those patients have to be put on immunosuppressants to suppress rejection in transplanted organs and reduce their defense against virus or bacterial infections (40). Therefore, SOT patients are at high risk of rapidly multiplying the virus and developing complications such as pneumonia, acute respiratory distress syndrome (ARDS), and sepsis when infected with COVID-19(41).

37 Anjan, Shweta, et al. "Is the Omicron variant truly less virulent in solid organ transplant recipients?." *Transplant infectious disease* 24.6 (2022): e13923.

38 Raja, Mohammed A., et al. "COVID-19 in solid organ transplant recipients: a systematic review and meta-analysis of current literature." *Transplantation Reviews* 35.1 (2021): 100588.

39 Kolla, Epiphane, et al. "COVID-19 Hospitalization in Solid Organ Transplant Recipients on Immunosuppressive Therapy." *JAMA Network Open* 6.11 (2023): e2342006-e2342006.

40 Duncan, Michael D., and David S. Wilkes. "Transplant-related immunosuppression: a review of immunosuppression and pulmonary infections." *Proceedings of the American Thoracic Society* 2.5 (2005): 449-455.

41 Kim, Min Young, Daniel C. Brennan, and Pali Shah. "General approach to the clinical care of solid organ transplant recipients with COVID-19 infection: management for transplant recipients." *Current transplantation reports* 7 (2020): 366-378.

The conclusion is not drawn in light of the facts. The severity instances typically occur in the Omicron period, according to Tables 1 and 2.

- The number of severe COVID-19 cases was much larger during the Omicron period compared to the Delta period, but the proportion of severe cases among confirmed cases was higher during the Delta period. In order to clarify the findings, we changed the sentence as follows.

**Before revision**

In conclusion, we found that the severity of COVID-19 infection was much higher for the Delta variant compared to the Omicron. Several risk factors such as vaccination, income level, and underlying disease including mental disorders differentially affected the development of critical COVID-19 illness between Delta and Omicron period. Closed monitoring of a wide range of risk factors for critical illness is warranted as new variants continuously emerge during the pandemic.

**After revision**

In conclusion, we found that the absolute number of severe COVID-19 cases was much larger during the Omicron period compared to the Delta period, but the proportion of severe cases among confirmed cases was higher during the Delta period. Several risk factors such as vaccination, income level, and underlying disease including mental disorders differentially affected the development of critical COVID-19 illness between Delta and Omicron period. Closed monitoring of a wide range of risk factors for critical illness is warranted as new variants continuously emerge during the pandemic.

Reviewer #2: Overall, this study evaluated the clinical characteristics of Covid-19 patients in Korea, and examined the association between severe COVID-19 cases and underlying health conditions during the Delta and Omicron period. The authors also acknowledged some limitations of the study (line 329-353). Thus, I have only minor comments:

1) Line 80: the abbreviation (CDC) should be used because it has been defined in line 75.

- Thanks for your detailed check. As your suggested, we used the abbreviation as follows.

**Before revision**

A review article by the Centers for Disease Control and Prevention of the United States reported the following were associated with the development of critical COVID-19 cases among adult patients: asthma, cancer, cerebrovascular disease, chronic kidney disease, chronic lung diseases, chronic liver diseases, cystic fibrosis, diabetes mellitus, disabilities, HIV (Human immunodeficiency virus), mental health conditions, neurologic conditions, obesity, physical inactivity, and so on.

**After revision**

A review article by the CDC of the United States reported the following were associated with the development of critical COVID-19 cases among adult patients: asthma, cancer, cerebrovascular disease, chronic kidney disease, chronic lung diseases, chronic liver diseases, cystic fibrosis, diabetes mellitus, disabilities, HIV (Human immunodeficiency virus), mental health conditions, neurologic conditions, obesity, physical inactivity, and so on.

2) Table 1: change “from diagnosis death” to “from diagnosis to death”.

- As you commented, we added “to” in the Table1 as follows.

**Before revision**

Time intervals, from diagnosis death within 1 month [median (IQR), days]

**After revision**

Time intervals, from diagnosis to death within 1 month [median (IQR), days]

3) Table 1, the time interval from diagnosis to death in Omicron is shorter than Delta, although the time interval from diagnosis to severe and critical are shorter in Delta and Omicron. Can the authors elaborate the findings in the discussion section?

- We assumed that such findings might be associated with the complex effect of virulence of the variants and capacity of healthcare system, but still further analysis is warranted. As you suggested, we tried to provide more plausible explanation for such findings as follows.

**Before revision**

We found that the time interval from diagnosis to severe or critical illness was longer during Omicron variants compared to Delta variants. This indicator reflects not only the characteristics of the variant itself such as virulence, but also the healthcare system contexts. The shorter period from diagnosis to developing a critical illness, which is less than six days during the Delta period, indicates the significant toxicity during that period compared to the typical onset time for general pneumonia. In contrast, during the Omicron period, the exponential increase in confirmed cases may result in delays in testing and hospitalization. However, further research is needed because it is difficult to determine whether the prolonged time to critical illness during the Omicron period was a delay in response after infection or a delay in medical utilization due to the nature of the claim data.

**After revision**

We found that the time interval from diagnosis to severe or critical illness was longer during Omicron variants compared to Delta variants. This indicator reflects not only the characteristics of the variant itself such as virulence, but also the healthcare system contexts. The shorter period from diagnosis to developing a critical illness, which is less than six days during the Delta period, seems to be associated with higher virulence of the Delta variant. However, the time interval from diagnosis to death during the Omicron dominant period was shorter than that during the Delta period. This is likely because the proportion of critical cases in the population over 80 was significantly higher during the Omicron period (44.09%) than during the Delta period (26.54%), and those elderly patients in a critical condition rapidly deteriorated partly due to underlying health conditions and incapacity of healthcare system overwhelmed by the Omicron epidemic, in spite of the low virulence of the Omicron variant. Further studies to clarify the reasons should be followed.

4) Table 2 and 3: how about the underlying HIV infections? It is strange that with the large number of data, it can’t capture the underlying HIV status, although it is mentioned in line 83.

- As for HIV* infection, the prevalence was very so low in South Korea that we could not provide separate estimates; according to our claim dataset, the number of confirmed patients with HIV infection was approximately 121 in the Delta period and 3351 in the Omicron period, of which the number of critical illnesses with HIV infection was 3 in the Delta period and 10 in the Omicron period.

*HIV definition: ICD-10 code “'B20”, “B21”, 'B22”, “B23”, “B24”, “Z21”

5) Line 288 and 289: describing virus with “less toxic” is not common. Please use another term, for example less virulent or less severe. It is also applied for the whole manuscript.

- As you pointed out, we replace the terms as follows.

**Before revision**

We observed a higher risk for critical illness among the elderly population during the Omicron period compared to the Delta period. Although the Omicron variant was less toxic, resulting in lower hospitalization and mortality rates, our study showed a stronger effect of mild toxicity in the 20-39 age group during the Omicron period, resulting in a relatively increased risk of critical illness in the elderly.

The shorter period from diagnosis to developing a critical illness, which is less than six days during the Delta period, indicates the significant toxicity during that period compared to the typical onset time for general pneumonia. In contrast, during the Omicron period, the exponential increase in confirmed cases may result in delays in testing and hospitalization. However, further research is needed because it is difficult to determine whether the prolonged time to critical illness during the Omicron period was a delay in response after infection or a delay in medical utilization due to the nature of the claim data.

**After revision**

We observed a higher risk for critical illness among the elderly population during the Omicron period compared to the Delta period. Although the Omicron variant was less virulent, resulting in lower hospitalization and mortality rates, our study showed a stronger effect of mild virulence in the 20-39 age group during the Omicron period, resulting in a relatively increased risk of critical illness in the elderly.

The shorter period from diagnosis to developing a critical illness, which is less than six days during the Delta period, seems to be associated with higher virulence of the Delta variant. However, the time interval from diagnosis to death during the Omicron dominant period was shorter than that during the Delta period. This is likely because the proportion of critical cases in the population over 80 was significantly higher during the Omicron period (44.09%) than during the Delta period (26.54%), and those elderly patients in a critical condition rapidly deteriorated partly due to underlying health conditions and incapacity of healthcare system overwhelmed by the Omicron epidemic, in spite of the low virulence of the Omicron variant. Further studies to clarify the reasons should be followed.

6) In the discussion, I would suggest including discussion about the association between pulmonary tuberculosis (TB) and Covid-19 severity. I think TB is the only infectious disease examined in this study?

- . As you suggested, we added the finding about the association between TB and the severity of COVID-19 in the Discussion section as follows.

**Before revision**

None.

**After revision**

The association between pulmonary tuberculosis (TB) and the severity of COVID-19 is not yet fully understood, with conflicting findings from different studies(47-50). A rapid systematic review and meta-analysis found that tuberculosis was associated with a 2.10-fold increased risk of severe COVID-19 disease, although the statistical difference was not significant(51). However, other studies have reported mixed findings, with some suggesting an increased risk of mortality in patients with underlying tuberculosis(52), while others indicate that patients with TB may have a less severe course of COVID-19 and a lower risk of mortality(53). A study comparing the clinical characteristics of patients with COVID-19/TB and patients with COVID-19 only found that female patients with TB were more likely to have severe COVID-19(49). On the other hand, a population-based, dynamic cohort study using national insurance claims databases found that having COVID-19 pneumonia was strongly associated with a higher hazard of detectable active pulmonary tuberculosis(54). Our study found that the risk of severe illness was significantly increased in cases with TB during the Omicron period (Odds Ratio: 1.18 [95% CI: 1.01, 1.37]), but not during the Delta period. Further research, including larger multicenter studies, is needed to determine the factors influencing mortality and severity in the COVID-19/TB cohort.

47 Stochino, Claudia, et al. "Clinical characteristics of COVID-19 and active tuberculosis co-infection in an Italian reference hospital." *European Respiratory Journal* 56.1 (2020).

48 Oh, Tak Kyu, and In-Ae Song. "Impact of coronavirus disease-2019 on chronic respiratory disease in South Korea: an NHIS COVID-19 database cohort study." *BMC pulmonary medicine* 21 (2021): 1-9.

49 Tamuzi, Jacques L., et al. "Implications of COVID-19 in high burden countries for HIV/TB: A systematic review of evidence." *BMC infectious diseases* 20.1 (2020): 1-18.

50 Wang, Yadong, et al. "An updated meta‐analysis on the association between tuberculosis and COVID‐19 severity and mortality." *Journal of medical virology* 93.10 (2021): 5682.

51 Gao, Ya, et al. "Association between tuberculosis and COVID‐19 severity and mortality: a rapid systematic review and meta‐analysis." *Journal of medical virology* 93.1 (2021): 194.

52 Prevention CfDCa. Brief Summary of Findings on the Association Between Tuberculosis and Severe COVID-19 Outcomes 2021 [Available from: <https://www.cdc.gov/coronavirus/2019-ncov/science/science-briefs/pdf/h-tb-review_final.pdf>.

53 Parolina, Liubov, et al. "Clinical characteristics of COVID-19 in patients with tuberculosis and factors associated with the disease severity." *International Journal of Infectious Diseases* 124 (2022): S82-S89.

54 Kumwichar, Ponlagrit, and Virasakdi Chongsuvivatwong. "COVID-19 pneumonia and the subsequent risk of getting active pulmonary tuberculosis: a population-based dynamic cohort study using national insurance claims databases." *Eclinicalmedicine* 56 (2023).
